# The Cheating Cell: How Evolution Helps Us Understand and Treat Cancer

**DOI:** 10.1093/emph/eoaa047

**Published:** 2020-11-25

**Authors:** David M Reese

**Affiliations:** Research and Development, Amgen Inc., One Amgen Center Drive, Thousand Oaks, CA 91320, USA

In a landmark paper published in 2000, the cancer biologists Douglas Hanahan and Robert Weinberg synthesized decades of investigation in tumor biology to identify six core capabilities acquired by cancer cells during the development and progression of human malignancies. According to their schema, the hallmarks of cancer include sustained proliferation, evading growth suppressors, resisting cell death (apoptosis), enabling replicative immortality, inducing angiogenesis (formation of new blood vessels) and activating invasion and metastasis [1]. Later updated in a follow-up publication to include common tumor characteristics such as abnormal metabolism and the ability to evade the immune system [2], the hallmark papers are now among the most cited in the history of cancer research and have provided a set of organizing principles for both basic and clinical investigators. 

Athena Aktipis, in her book *The Cheating Cell: How Evolution Helps Us Understand and Treat Cancer*, urges us to go beyond the hallmarks to understand cancer—and, in so doing, to conceive of new ways to prevent and treat the disease. She makes a compelling case that insights from evolutionary biology and ecology can complement the descriptive phenomenology that has characterized most cancer research.

The core argument of *The Cheating Cell* is contained in the title of the book: cancer, at the most basic level, can be regarded as ‘the ultimate form of cellular cheating’. Normally, the trillions of cells in our bodies harmoniously cooperate, following what Aktipis calls the multicellularity playbook. Normal cells do not divide uncontrollably, they self-destruct if they become threatening, they share resources, they perform a defined job, and they take care of their local environment. In other words, normal cells do not have any of the hallmarks of cancer, and they occupy a defined niche in a broader ecosystem.

Aktipis, a professor at Arizona State University and cofounder of the International Society for Evolution, Ecology and Cancer, sees breakdown of this cellular cooperation as the fundamental characteristic of the cancer cell. The molecular defects that comprise the Hanahan and Weinberg hallmarks of cancer, in this view, are specific mechanisms that cancer cells use to become non-cooperators, but defining cancer more broadly as a failure of cooperation provides a comprehensive framework to understand the disease, one that can unify perspectives from tumor genetics, immunology, cell biology and comparative biology.

Artfully written in non-technical prose, *The Cheating Cell* will appeal to both educated lay readers and professionals looking for an introduction to evolutionary concepts in cancer biology. In the first part of the book, Aktipis identifies cancer as a natural consequence of multicellularity. Over the course of a human lifetime, there are a staggering number of cell divisions, and, given the imperfect fidelity of molecular editing, genetic mistakes occur. An accumulation of mutations in so-called driver genes—roughly four to seven, on average, for common tumors such as lung and prostate cancer—eventually produces a fully malignant cell. This is why most cancers increase in incidence with age. It simply takes time to develop the requisite number of mutations, in the right combination, for cancer to develop.

Subsequent chapters lay out the argument for viewing cancer through an evolutionary lens. Aktipis first notes that a population of cancer cells meets the basic conditions for evolution through natural selection. They possess genetic variability, their mutations are heritable, and the traits these variations produce confer differential fitness, both as a tumor develops and under the selection pressure of treatments such as chemotherapy. These are simple evolutionary concepts, but are too often forgotten in the hyperspecialized world of cancer research, and Aktipis does well to remind us of their importance.

Aktipis extends her argument by introducing the notion of evolutionary trade-offs in cancer biology. The cancer cell, characterized by the hallmarks, proliferates uncontrollably, fails to undergo programmed cell death and evades the immune system. According to Aktipis, each of these features reflects an evolutionary trade-off made by multicellular organisms. Rapid cellular proliferation is essential in development, fertility and wound healing, for instance, but the same molecular mechanisms that drive proliferation in these settings can be subverted by the cancer cell. Likewise, elimination of genetically damaged cells must be finely balanced. If the mechanisms of suppression are too vigorous, premature aging will occur as normal cells are inappropriately induced to under apoptosis, whereas loss of surveillance and DNA repair mechanisms leads to cancer. In fact, the tumor suppressor gene *TP53*, which plays a central role in regulating the cell cycle, apoptosis and genomic stability, is mutated in more than 50% of human cancers, making it the single most common genetic alteration in tumor cells and underscoring the importance of active tumor suppression in preventing the development of the cancer cell. Aktipis nicely harmonizes the cellular and organismal perspective in leading the reader through this biology.

*The Cheating Cell* rounds out its argument in favor of an evolutionary framework by taking the reader through examples of cancers in the animal and plant world, such as crested cacti, whose fasciations can be regarded as a sort of tumor. There are excursions into transmissible facial tumors in Tasmanian devils, a discussion of why elephants appear to be more resistant to the development of cancer (their cells have multiple copies of the tumor suppressor *TP53*), and an analysis of antleromas in deer, which presumably arise due to selection pressure that favors the rapid growth of large antlers, thereby creating increased susceptibility to cancer. All of these cases buttress the thesis that cancer is, at basis, a failure of cellular cooperation.

As a medical oncologist, I was most looking forward to the conclusion of the book, a discussion of how viewing cancer from an evolutionary perspective can inform our approach to treatment and the development of new drugs. Aktipis spends a number of pages describing adaptive therapy, an approach that aims to vary the doses and combinations of drugs over time to control—but not eradicate—a tumor, much as farmers employ pest management strategies that use lower doses of pesticides to maintain pest infestations at an acceptable level. Adaptive therapy in various forms has been tried in a number of tumors, but it is not yet clear if it provides an advantage over standard approaches. 

More promising is the discussion of Evo and Eco Indexes, ways of categorizing tumors based on their evolutionary (genetic diversity, rate of genetic change) and ecological (resources such as blood supply, immune system activity) features. In the last few decades the development of targeted therapies and immunotherapy has transformed the treatment of many cancers, and there is enormous opportunity in marrying what we have learned at a molecular level about the response and resistance of tumors to these new treatments with the framework that Aktipis supplies in *The Cheating Cell*. That marriage could indeed lead to the future that Aktipis envisions, one in which cancer is eradicated when possible and controlled when it cannot be eliminated—a future when the cheating cell gets its comeuppance. 
